# Deployment and uptake of COVID-19 vaccines for refugees and migrants in Ecuador

**DOI:** 10.3389/fpubh.2025.1655392

**Published:** 2025-11-04

**Authors:** Cheryl Martens, Taymi Milan, María Belén Mena Ayala, Enrique Teran, Pierina Benavente, N. T. Tran, Karl Blanchet

**Affiliations:** ^1^Instituto de Estudios Avanzados en Desigualdades, Universidad San Francisco de Quito, Quito, Ecuador; ^2^University of Galway, Galway, Ireland; ^3^Facultad de Ciencias Médicas, Instituto de Biomedicina Universidad Central del Ecuador, Quito, Ecuador; ^4^Pandemic Centre, Department of Global Public Health and Primary Care, University of Bergen, Bergen, Norway; ^5^Faculte de Medecine, Universite de Geneve, Geneva, Switzerland; ^6^Australian Center for Public and Population Health Research, Faculty of Health, University of Technology Sydney, Sydney, NSW, Australia

**Keywords:** vaccine access, discrimination, migrants, refugees, human rights

## Abstract

This study examines access and uptake of COVID-19 vaccines among refugees and migrants in Ecuador, including those with regular and irregular migration status. Conducted in Quito, Manta, and Huaquillas with 344 participants, the article reports on the survey data to assess vaccination access, barriers, and enablers. Findings show that 94% of respondents received at least one vaccine dose, despite 69% having irregular status. However, gaps remained in second and booster dose uptake, which was linked to misinformation and administrative barriers such as lack of documentation, discrimination and stigma, especially from healthcare and security personnel at vaccine sites. Key facilitators included receiving support from non-governmental organizations, mobile health brigades, and pressure from international organizations. The study concludes that although Ecuador made vaccines accessible to migrants, systemic challenges, such as data gaps, xenophobia, and insufficient outreach, hindered equitable coverage and limited the rights of migrants and refugees. Improved communication, flexibility in relation to documentation are recommended to ensure equitable access vaccines.

## 1 Introduction: context of migration and COVID-19 in Ecuador

There has been an unprecedented increase in migratory flows in Latin America, demonstrating that Ecuador has one of the highest levels of migration in the region, with more than 588 900 Venezuelan migrants, in relation to its small population of just over 18 million. The COVID-19 pandemic and the complex economic situation brought new challenges that affected vulnerable populations, including migrants. This population faced structural and social barriers that limit timely access to vaccination, a finding that has been demonstrated on a global level, and recorded within Latin America as well as Europe, where migrants face major barriers to vaccination, including language, legal restrictions (1).

During the pandemic, migrants in Ecuador reported increased discrimination, due to misinformation and stereotypes, resulting in the holding migrants responsible for the spread of the virus, impacting the risk of infection, mortality, and access to timely health care (2, 3).

In the context of COVID-19, the Ecuadorian government initiated its national vaccination plan “Vacunarse” during the final months of the government of president Moreno, in December 2020, followed by the “Plan 9/100”, at the beginning of the first term of the president Lasso government on May 24th, 2021. Under the government of Lasso, the promise was to vaccinate 9 million people within the first 100 days of government. It is essential to note that Ecuador was the first country in the Andean region to implement a vaccine program specifically designed for its migrant population (2–5). In comparison with other countries in the region, Bolivia, Peru, and Paraguay lagged behind Ecuador (6). Comparative analyses suggest that earlier rollout, stronger supply chains, and broader eligibility criteria contributed to the faster uptake in higher-performing countries (6, 7).

In the case of Ecuador, a quarantine was imposed to contain the COVID-19 pandemic. However, the closure of borders and their militarization resulted in the suspension of all official migration processes, including the administrative process for applying for refugee status.

Several studies demonstrate the negative impact of these policies on the lives of people migrating to Ecuador, as access to food and services was reduced throughout the pandemic (8, 9). A significant result of the border closures was an increase in undocumented crossings to Ecuador throughout the pandemic. Limited official numbers and the dismantling of humanitarian aid and registration of migrant numbers by the Red Cross and other international ceased during the pandemic and has resulted in a complex system of intersectoral reporting and statistical estimations based mainly on access to the services of non-governmental and intergovernmental organizations and analyzed by the interagency working group for migrants and refugees (GTRM, by its acronym in Spanish “Grupo de Trabajo para Refugiados y Migrantes”).

An impediment to measuring the impact of COVID-19 policies on migrants is the unevenness of governmental statistical record-keeping concerning the migrant population. According to the National Institute for Statistics and Census (INEC), as of March 21, 2022, the Ecuadorian Institute for Statistics reported that most registered foreign residents in Ecuador are of Colombian nationality (10). Of the 381,507 migrants in the country, 191,537 (50.2%) are of Colombian origin. In second place are US residents with a total of 26,386 migrants. However, this information is not considered to be accurate by international organizations, as the GTRM, an intersectoral working group for the articulation of actions agreed upon by various partners to address the protection, assistance, and integration needs of refugees and migrants from Venezuela in Peru, estimates that there are ~508,935 Venezuelans in Ecuador (11). This estimate is based on registering entry and exit from the country and analyzing, monitoring, tracking, and characterization of flows at the border.

During the pandemic in December 2021, more than 71,550 persons were registered in Ecuador as refugees. Of the refugee population, more than 97% are of Colombian origin.

## 2 Materials and methods

The overall objective of the study is to produce evidence on the barriers and facilitators to COVID-19 vaccination for refugees, migrants in regular situations (MIRS), and migrants in irregular situations (MIIS) in Ecuador, who are impacted by large-scale migration, and to provide an estimate of COVID-19 vaccination coverage among these three groups.

The specific objectives are to:

1. Understand the status of COVID-19 vaccine access and uptake, knowledge and perspectives, attitudes and practices, challenges, barriers, and enablers in refugees and migrants' access to vaccines regardless of their status in Ecuador.
2. Estimate the proportion of refugees and migrants reached by COVID-19 vaccination campaigns in Ecuador.

**Figure 1:**
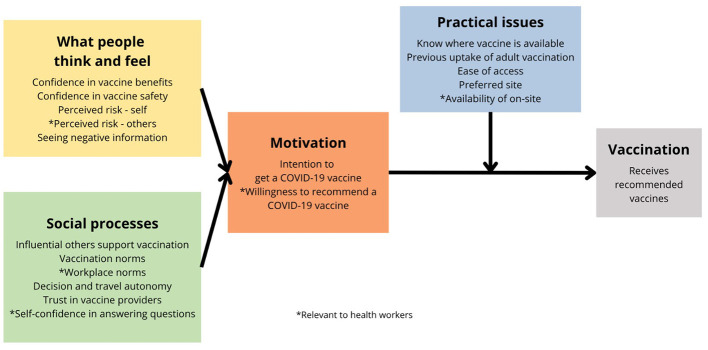
The behavioral and social drivers (BeSD) of COVID-19 vaccination framework.

This study on COVID-19 vaccination uptake in Ecuador is part of a series of country-level studies grounded in the WHO Health Systems Framework, with its six system building blocks, and the Behavioral and Social Drivers (BeSD) of COVID-19 Vaccination Framework (Figure 1) (12, 13, 25, 26). The six system building blocks are leadership and governance, healthcare financing, health workforce, medical products and technologies, information and research, and service delivery. The WHO Health System Framework not only informs the design and content of our surveys but also enhances the actionability of our findings regarding health system strengthening. The BeSD of the COVID-19 Vaccination Framework enables careful consideration of how individual, social, and structural determinants interact to influence willingness to vaccinate and vaccination uptake.

The overall study consisted of two phases, corresponding to each of the objectives. In the first stage, a document analysis was conducted, along with key informant interviews, and in the second stage, the application a survey. The findings reported in the present article pertain to the survey data on to migrants and refugees, facilitators and barriers to vaccine access.

### 2.1 Survey instrument

The quantitative survey was designed based on the following guidelines and validated resources: (1) WHO and UNICEF (14); (2) Data for action: achieving high uptake of COVID-19 vaccines; (3) United States Centers for Disease Control and Prevention. Vaccine confidence survey, question bank (15); (4) United States Centers for Disease Control and Prevention (16); (5) COVID-19 vaccine confidence rapid community assessment guide; (6) WHO Regional Office for Africa (17). Social and behavioral insights: COVID-19 data collection tool for Africa; (7) WHO and UNICEF (18). Monitoring COVID-19 vaccination: considerations for the collection and use of vaccination data; (8) IOM and National Statistics Service of the Republic of Armenia (19). Report on household survey on migration in Armenia; (9) United Nations Expert Group on Migration Statistics (20); (10) Standard questions on international migration: Guidance note for the use in population censuses and household surveys. The interview guides were translated into Spanish, the main language spoken by migrants and refugees in Ecuador.

### 2.2 Sampling and data collection

For the survey, purposive sampling was used with a sample of 344 people distributed in the cities Quito (*n* = 194), the capital city and home to the highest estimated migrant population, the coastal city of Manta (*n* = 72), and the border city of Huaquillas (*n* = 78).

These cities were selected, given that they concentrate a high proportion of migrant population. The city of Quito has the highest level of migrant population; for that reason, the sample of Quito is proportionately higher to the other two cities sampled. Participants included all refugee, MIRS, or MIIS communities who qualified for the COVID-19 vaccine in Ecuador. Recruitment took place in collaboration with local NGOs with the highest percentage of migrant and refugee service users in each of the three cities. Recruitment was initially made through announcements on social media within migrant organizations migrant-focused foundations and associations that provide support and community engagement for refugees and migrants. While this approach facilitated access to diverse migrant groups, it may have introduced selection bias, as individuals not affiliated with such organization that may be among the most marginalize, could have been excluded.

To enhance representativeness and minimize this bias, the research team employed a referral (snowball) sampling method, allowing each respondent to refer other migrants within their networks. Significantly, none of the individuals who were invited to participate declined to do so. Nevertheless, the total number of individuals approached and those who declined participation were not systematically recorded, which we recognize as a limitation.

A small gift of food was given to each participant who decided to participate. Only those participants who agreed to participate and provided informed consent were included in the survey. No participant was excluded based on gender, race, religion, ethnicity, or other characteristics.

The survey was conducted in the cities of Quito, Manta, and Huaquillas, in collaboration with local NGOs that work with migrants and refugees. These organizations carried out a call for participation in the survey among their service users. The data collection process took place in the city of Quito from May 30th to June 2nd, 2022, at the Asociación Civil Chamos Venezolanos en Ecuador, where the migrant population and data collection were conducted at the organization's premises. Subsequently, data collection took place from July 27, 2022, to July 30, 2022, in the city of Huaquillas, in collaboration with Asociación Venezolanos en Exterior. The data collection process was conducted at the organization's premises. Finally, the data collection process was conducted in Manta with the support of the Organization La Chama de Manta from August 6, 2022, to August 8, 2022, during which interview spaces were provided.

### 2.3 Data collection

An online questionnaire designed using Kobo Toolbox, facilitated data collection for in-person survey interviews. The questionnaire's contents were finalized in consultation with members of the technical committee at USFQ (see Annex I for the survey questionnaire).

For the data collection, respondent answers to interview questions were simultaneously loaded on an online database by the interviewers as they conducted the interviews. Information was collected through in-person surveys and entered on a survey platform via mobile telephones.

The manager verified the accuracy and completeness of the entered data at various levels and stages. Only the research team had access to the server to download and check data quality.

The adults participating in the study gave informed written consent. All participants were over 18 years of age and informed about the right to refuse or withdraw from the survey at any time.

Data was gathered in the following areas: (1) Demographics; (2) Migration status; (3) COVID-19 infection; (4) Risks; (5) Infection status; (6) Testing; (7) Hospitalizations; (8) Preventive measures; (9) COVID-19 vaccination coverage, knowledge, access, and funding; (10) Behavior in context of infection and vaccination; (11) Motivation and Barriers.

### 2.4 Ethics and quality monitoring

The survey design, sampling strategy, instruments, and analytical plans were reviewed and approved by the Universidad San Francisco de Quito Ethics Committee. The confidentiality of all collected data was given high priority at every stage of data handling. The research participants were informed about the purpose, methods, benefits, and intended uses of the research. Informed verbal consent was obtained from the research subjects. Respondents were free to stop interviews at any time or skip any questions they did not want to answer. They had the right to ask questions at any point before, during or after the interview. Individual names and personal information of respondents were kept confidential, and personal identifiers were not used in any form of reporting. Datasets were also kept anonymous for analysis. All data files were saved in password-protected files.

Steps were taken to ensure the quality of data collection. Team leaders managed the teams and monitored activities, and reviewed all completed questionnaires for completeness and inconsistencies before leaving the village. Team leaders also did spot checks of data forms and provided guidance and supportive supervision to the field teams through continuous reinforcement of good practices. The challenges faced by teams were discussed, solutions developed, and feedback provided to team leaders.

## 3 Results

### 3.1 Participant profiles

The sample size for this study was 344 respondents, of whom 90.41% self-identified as Venezuelan. 5.81% of respondents reported their nationality to be Colombian, followed by Cuban nationals (1.5%) and 2.5% of other nationalities, mainly from Latin America and Africa. The survey was conducted in the capital city of Quito (Pichincha), with a sample size of 194, as well as in the cities of Manta in the province of Manabí (*n* = 72) and the city of Huaquillas in El Oro (*n* = 78).

Respondents were between 18 and 49 years of age, with the majority (49% of respondents) in the 30–49 age group, followed by 33% in the 18–29 age group. In contrast to these two age groups, which could be considered as young adults and adults, a minority of 3% of respondents were over 65 years of age. In the total sample (344 respondents), 53% were female, 46% male and 1% identified as “other”. The higher percentage of female respondents supports the findings of other surveys of migrants in Ecuador (12, 13).

Regarding the level of education, 66% of respondents reported having completed secondary studies or higher. A total of 37% of the sample reported having completed secondary education, 27% possessed a university undergraduate degree, and 2% of respondents had completed a postgraduate degree.

In terms of length of time in Ecuador, 60.47% of the sample reported being in the country for between 1 and 5 years, followed by stays of < 1 year. Of those surveyed, 69% were MIIS, meaning that they do not possess a visa or are in the process of obtaining one, and 5% were refugees.

### 3.2 Self-reported COVID-19 infection

Analysis of self-reported COVID-19 infection prevalence revealed differentiated patterns according to gender and migratory status (Table 1). Among the regular migrant population (*n* = 90), a significant gender disparity was identified (χ^2^ = 8.31, *p* = 0.016). Men reported an infection prevalence of 43.3% (26/60), significantly higher than that observed in women at 13.3% (4/30), resulting in an absolute difference of 30.0 percentage points (95% CI: 9.2% to 50.8%) favoring lower infection rates in women.

**Table 1:** Self-reported COVID-19 infection prevalence by gender and migratory status.

**Migratory status**	**Gender**	**Infected *n* (%)**	**Not infected *n* (%)**	**Does not know/ recall *n* (%)**	**Total**	Difference in proportions^*^% (95% CI)	***p*-value^†^**
Regular	Female	4 (13.3)	20 (66.7)	6 (20.0)	30	30.0 (9.2–50.8)	0.016
	Male	26 (43.3)	26 (43.3)	8 (13.3)	60	–	–
	Subtotal	30 (33.3)	46 (51.1)	14 (15.6)	90	–	–
Irregular	Female	44 (31.7)	81 (58.3)	14 (10.1)	139	2.7 (−10.4 to 15.8)	0.914
	Male	33 (34.4)	45 (46.9)	18 (18.8)	96	–	–
	Subtotal	77 (32.8)	126 (53.6)	32 (13.6)	235	–	–
Refugee	Female	3 (37.5)	4 (50.0)	1 (12.5)	8	0.0 (−43.7 to 43.7)	1.000
	Male	3 (37.5)	5 (62.5)	0 (0.0)	8	–	–
	Subtotal	6 (37.5)	9 (56.3)	1 (6.3)	16	–	–
Overall total		113 (33.1)	181 (53.1)	47 (13.8)	341	–	–

^*^Difference calculated as proportion in males minus proportion in females (positive values indicate higher prevalence in males).

^†^Pearson's Chi-square test or Fisher's exact test as appropriate.

CI, confidence interval at 95%.

*n* = 3 participants who reported gender as “Other” were excluded from analysis (2 irregular, 1 refugee).

In contrast, no statistically significant gender differences were identified in either irregular migrant or refugee populations. Among irregular migrants (*n* = 235), prevalence was 31.7% (44/139) in women and 34.4% (33/96) in men, with a difference of 2.7 percentage points (95% CI: −10.4% to 15.8%, *p* = 0.914). In the refugee population (*n* = 16), both genders presented identical prevalence rates of 37.5% (3/8 in each group; difference: 0.0%, 95% CI: −43.7% to 43.7%, *p* = 1.000), although the limited sample size substantially reduces statistical power to detect differences.

Notably, between 6.3% and 15.6% of participants, depending on their migratory status, did not recall or were unaware of whether they had been infected, with this proportion being higher among regular migrants (15.6%, 14/90) compared to irregular migrants (13.6%, 32/235) and refugees (6.3%, 1/16).

### 3.3 Vaccination by gender and migration status

Irrespective of migratory status, the vast majority of respondents had been offered a COVID-19 vaccination. A total of 323 respondents, corresponding to 94% of the sample, reported having received a COVID-19 vaccination. Furthermore, a large proportion of the respondents were in the group with irregular migration status with 69%, followed by persons with regular status with 27% and refugees, respectively.

Seventy-six percent of the respondents who were vaccinated had received two or more doses of the virus vaccine, including one dose of CanSino or two or more doses of other vaccines. Sixteen percent reported having received only partial doses of the vaccine, and 7% had not received full doses. Regardless of the population's migration status, most reported receiving full doses of the COVID-19 vaccine.

Analysis of COVID-19 vaccination offer revealed gender-specific disparities according to migratory status (Table 2). In the overall sample (*n* = 341), 93.8% (320/341) reported having received a vaccination offer, with an overall coverage of 97.7% (167/171) in women compared to 90.0% (153/170) in men.

**Table 2:** COVID-19 vaccination offer by gender and migratory status.

**Migratory status**	**Gender**	***N***	**Vaccine offered *n* (%)**	**Vaccine not offered *n* (%)**	Difference in proportions^*^% (95% CI)	**OR (95% CI)^†^**	**p-value^‡^**
Regular	Female	30	30 (100.0)	0 (0.0)	3.3 (−3.1 to 9.7)	NC	0.548
	Male	60	58 (96.7)	2 (3.3)	–	–	–
	Subtotal	90	88 (97.8)	2 (2.2)	–	–	–
Irregular	Female	139	133 (95.7)	6 (4.3)	7.2 (0.6–13.8)	2.87 (1.04–7.91)	0.034
	Male	96	85 (88.5)	11 (11.5)	–	–	–
	Subtotal	235	218 (92.8)	17 (7.2)	–	–	–
Refugee	Female	8	8 (100.0)	0 (0.0)	25.0 (−7.0 to 57.0)	NC	0.467
	Male	8	6 (75.0)	2 (25.0)	–	–	–
	Subtotal	16	14 (87.5)	2 (12.5)	–	–	–
Overall total		341	320 (93.8)	21 (6.2)	–	–	–

^*^Difference calculated as proportion in females minus proportion in males (positive values indicate higher offer in females).

^†^Odds ratio calculated with males as reference category (OR > 1 indicates higher odds in females).

^‡^Pearson's Chi-square test or Fisher's exact test as appropriate.

NC, not calculable due to presence of cells with zero values preventing OR calculation. OR, odds ratio; CI, confidence interval at 95%.

*n* = 3 participants who reported gender as “Other” were excluded from analysis (2 irregular, 1 refugee).

Among the irregular migrant population (*n* = 235), a statistically significant gender difference was identified (χ^2^ = 4.48, *p* = 0.034). Women presented higher offer coverage at 95.7% (133/139) compared to 88.5% (85/96) in men, resulting in an absolute difference of 7.2% points (95% CI: 0.6% to 13.8%). Odds ratio analysis confirmed that irregular migrant women had 2.87 times higher odds of receiving a vaccination offer compared to men (OR = 2.87, 95% CI: 1.04–7.91), with this association being statistically significant.

No statistically significant differences were observed in regular migrant populations (100.0% in women vs. 96.7% in men, *p* = 0.548) or refugees (100.0% vs. 75.0%, *p* = 0.467). In the case of refugees, although a considerable numerical difference of 25.0 percentage points was observed, the limited sample size (*n* = 16) substantially reduces statistical power to detect significant differences, which may explain the absence of statistical significance despite the magnitude of the observed difference.

These findings suggest that gender inequities in access to COVID-19 vaccination offer are specific to the migratory context, being particularly evident in the irregular migrant population where men experience additional barriers to accessing immunization offers.

### 3.4 Timing and brand of first-time vaccinations by gender and migration status

Most participants (81%) received their first dose between July and September 2021, with 50% being female and 50% being male. Regarding the migratory status of those vaccinated between July and December, 44% had regular status, 44.5% had irregular status, and 10% had refugee status. The Sinovac brand of vaccine predominated (47%), followed by Pfizer (19%), Astrazeneca (15%), and 9% of respondents received the single-dose vaccine, CanSino. There was an additional 7% that received other vaccine brands (Sinopharm, Sputnik, J&J, or Moderna), and 3% didn't know/didn't remember the brand. In total, 83% of respondents had received the first dose in Ecuador, followed by 6.5% in Colombia and 6.2% in Venezuela.

The majority of participants (61%) received their second dose between July and September 2021, of which 50% were female and 50% were male. This period was followed by October to December, during which a total of 28% of respondents had been vaccinated. The Sinovac brand of vaccine predominated (51%), followed by Pfizer (24%), Astrazeneca (17%), and only one person received the single dose of CanSino (0.5%). There was an additional 3.5% that received other vaccine brands (Sinopharm, Sputnik, or Moderna), and 4% didn't know/didn't remember the brand. Ninety percent of the population vaccinated with the second dose received it in Ecuador, followed by 4% vaccinated in Colombia, and 4% in Venezuela.

Most participants received their booster dose between January and June 2022, with 48% being female and 52% male. The Sinovac brand of vaccine predominated (27%), followed by Pfizer (29%), AstraZeneca (36%), and 4% of respondents received the single-dose CanSino. There was an additional 2% that received other vaccine brands (Sinopharm, Sputnik), and 2% didn't know/didn't remember the brand. Ninety-four percent of the population vaccinated with the second dose received it in Ecuador, followed by 4% in Peru, and in equal magnitude Chile and Venezuela (1%).

### 3.5 Experience of the vaccination process

The majority of respondents (71%) had not heard or observed any comments about the vaccination process for foreign nationals against COVID-19. Indeed, of the 344 respondents, only 26% had heard something and 3% were unsure.

Of the 88 respondents surveyed who reported having seen or heard any comments, 18 were of regular migration status, 65 were of irregular status, and 5 were of refugee status. Among the types of actions observed and reported, the predominant ones were that documentation, such as a visa or identity card, was requested to administer the doses (33%) and that it was possible to be vaccinated despite not having a visa or any other legal document (18%).

Of these data, it should be emphasized that one person with regular migratory status heard that respondents without documentation would be deported if they went for vaccination. In addition, 5 respondents, 4 were MIIS and one refugee, heard that the vaccinations were only for Ecuadorians.

Respondents were asked if they perceived differences in the treatment for vaccination in relation to foreign citizens. However, 91% of respondents did not perceive any differences, while 6% said they perceived a difference, and 3% did not know or could not remember. Among the 19 respondents who perceived differences, 5 were MIRS, 12 MIIS, and 2 were refugees.

Similarly, respondents were asked if they had witnessed any form of discrimination during the vaccination process, and 93% of the surveyed population reported not having done so. Meanwhile, 5% of the surveyed population stated that the acts of discrimination were directed against them personally, and 2% against the migrant population. The acts of discrimination reported against this group of respondents were mainly by health personnel (54%), security personnel (25%) and local respondents (13%).

## 4 Discussion

In Ecuador, although the majority of the participants were migrants in irregular situations (MIIS), nearly all respondents in our study had been offered a COVID-19 vaccine. This reflects regional patterns, where Latin America achieved relatively high levels of vaccination compared with other low- and middle-income regions, although marked gaps remained in terms of booster coverage and equitable inclusion of vulnerable groups (19, 20).

Our findings showed that only 61% of respondents received a second dose and even fewer obtained boosters are consistent with evidence of undervaccination described in systematic reviews of migrant populations. Crawshaw et al. (1) identified key determinants—including misinformation, administrative hurdles, and lack of culturally tailored communication—that mirror the barriers reported in Ecuador. This suggests that vaccine uptake is not merely a function of supply, but also depends on addressing confidence, convenience, and the social determinants of health.

The Ecuadorian case also highlights broader global inequities in vaccine distribution. While high-income countries reached nearly 80% one-dose coverage by late 2023, low-income countries lagged behind at only 33% (21). Within Latin America, comparative analyses show that countries with stronger supply chains and more inclusive eligibility criteria achieved faster uptake, while others struggled with fragmented policy responses (19).

In Ecuador, once the availability of the vaccine was resolved bymid-2021, most of the migrants (81%) received their first dose. However, it is also important to note that the second dose was received only by 61%, as well as the booster. Something that might be related to misinformation or lack of proper communication campaigns, as the sanitary authority insisted there was enough inventory for all people living in the country. This was not, unfortunately, the same everywhere, as of 29 November 2023, it was estimated that 79.9% of the population in HICs had been vaccinated for COVID-19 with at least one dose, while in LICs only 32.8% of the resident population had received at least one shot (19).

The International Organization for Migration (IOM) reported that, when the vaccine rollout started, only regular migrants were included in vaccination campaigns in most countries (21), something that is also reported in our study. However, more recent updates from the UNHCR report that 162 countries have included refugees in their national COVID-19 vaccine plans; however, information about the inclusion of irregular migrants in the different local vaccination programs is scanty (22).

It has also been reported that refugees and migrants (particularly MIIS) may face multiple barriers to vaccination against COVID-19, including limited vaccine supply; low confidence in the benefits and safety of the vaccine; social influence and norms; lack of information on how to obtain vaccines; language barriers; complex registration processes and limited access to the web; and fear of arrest, detention, or deportation (23) but as mentioned previously, in Ecuador most of these issues also were reported. Some of those barriers were resolved quicker than others achieving a positive outcome. On the other hand, operational and administrative barriers (such as identification documents and residence permit) limited migrants in Ecuador access to vaccines as well reported by WHO (24).

Finally, stigma, discrimination, exclusion, and lack of access to health information and quality healthcare all represent additional barriers affecting access to this basic human right, not only in our sample, and other studies pertaining to migrants and refugees (24).

Migrants and refugees with irregular status in particular, continue to be among the most marginalized in vaccination programs. Although Ecuador adopted comparatively inclusive policies, our results confirm that barriers persisted, such as documentation requirements, discrimination by health and security staff, and misinformation. These obstacles echo findings from PAHO (6) and Crawshaw et al. (1), which emphasize the importance of targeted communication strategies, reduction of administrative obstacles, and collaboration with NGOs to ensure equitable vaccine access.

## 5 Conclusion

The analysis of the results of this research demonstrates several main issues concerning COVID-19 vaccine access and deployment in Ecuador, impacting the human rights of migrants and refugees in Ecuador, related especially to the initial lack of funding for vaccines, as well as the need for communication to counter xenophobia and misinformation.

The main conclusions are that there is a lack of accurate information gathering concerning vaccination records within Ecuador. Vaccine record keeping has improved since May 2021; however, there remain major gaps concerning the administration of vaccinations. These data gaps are related to insufficient training and oversight of staff at the vaccination centers concerning data classification as well as the procedures that ensure equal access to vaccines.

Discrimination by gatekeepers, such as security guards and healthcare workers was a barrier that made the implementation of universal vaccination programs difficult. Persons with disabilities and belonging to the LGBT+ population faced further discrimination, providing evidence that further research needs to be focused on how vaccine rollouts affect these vulnerable populations.

The lack of the possibility to publicly access stable and easy-to-access information about vaccination programs and misinformation makes it difficult for migrants and refugees to access vaccination points.

Initial low investment in the Ecuadorian health system and dependency on International Organizations for donations were tremendous barriers to facilitating access to vaccination, both for Ecuadorians and even more for those with regular as well as irregular status and refugees. The lack of support makes medium—to long-term planning difficult.

Access to vaccination centers and health brigades, which reached communities that were not accessing vaccination center sites, were the main facilitators in the vaccine roll-out.

Social pressure from non-governmental and multilateral organizations and improvements to the vaccine registration process for vaccines against COVID-19 have progressively facilitated migrants' access to the basic and constitutional right of everyone living in Ecuador to health.

Our study demonstrates outcomes broadly consistent with findings in Latin America and Europe, where vaccine accessibility improved but booster uptake lagged behind (1, 19). In line with regional evidence (20), our results highlight that equitable policies can reduce gaps in initial vaccine access, yet operational and social barriers persist. Compared to global analyses of vaccine inequity, which point to higher mortality in under-resourced regions (21, 22), Ecuador's relatively inclusive approach mitigated some of these risks, though gaps in booster coverage remain a shared challenge.

## Data Availability

The raw data supporting the conclusions of this article will be made available by the authors, without undue reservation.
